# Mobile Health Apps in Pediatric Obesity Treatment: Process Outcomes From a Feasibility Study of a Multicomponent Intervention

**DOI:** 10.2196/16925

**Published:** 2020-07-08

**Authors:** Sarah Browne, M-Tahar Kechadi, Shane O'Donnell, Mckenzie Dow, Louise Tully, Gerardine Doyle, Grace O'Malley

**Affiliations:** 1 School Public Health, Physiotherapy & Sports Science University College Dublin Dublin Ireland; 2 Insight Centre for Data Analytics School of Computer Science University College Dublin Dublin Ireland; 3 School of Sociology University College Dublin Dublin Ireland; 4 Division of Population Health Sciences School of Physiotherapy Royal College of Surgeons in Ireland Dublin Ireland; 5 UCD Michael Smurfit Graduate Business School University College Dublin Dublin Ireland; 6 W82GO Child and Adolescent Weight Management Service Children's Health Ireland at Temple Street Dublin Ireland

**Keywords:** childhood obesity, diet therapy, mHealth, mobile phones, smartphones, appetite, satiety, rate of eating, accelerometer, physical activity

## Abstract

**Background:**

Multicomponent family interventions underline current best practice in childhood obesity treatment. Mobile health (mHealth) adjuncts that address eating and physical activity behaviors have shown promise in clinical studies.

**Objective:**

This study aimed to describe process methods for applying an mHealth intervention to reduce the rate of eating and monitor physical activity among children with obesity.

**Methods:**

The study protocol was designed to incorporate 2 mHealth apps as an adjunct to usual care treatment for obesity. Children and adolescents (aged 9-16 years) with obesity (BMI ≥98th centile) were recruited in person from a weight management service at a tertiary health care center in the Republic of Ireland. Eligible participants and their parents received information leaflets, and informed consent and assent were signed. Participants completed 2 weeks of baseline testing, including behavioral and quality of life questionnaires, anthropometry, rate of eating by Mandolean, and physical activity level using a smart watch and the myBigO smartphone app. Thereafter, participants were randomized to the (1) intervention (usual clinical care+Mandolean training to reduce the rate of eating) or (2) control (usual clinical care) groups. Gender and age group (9.0-12.9 years and 13.0-16.9 years) stratifications were applied. At the end of a 4-week treatment period, participants repeated the 2-week testing period. Process evaluation measures included recruitment, study retention, fidelity parameters, acceptability, and user satisfaction.

**Results:**

A total of 20 participants were enrolled in the study. A web-based randomization system assigned 8 participants to the intervention group and 12 participants to the control group. Attrition rates were higher among the participants in the intervention group (5/8, 63%) than those in the control group (3/12, 25%). Intervention participants undertook a median of 1.0 training meal using Mandolean (25th centile 0, 75th centile 9.3), which represented 19.2% of planned intervention exposure. Only 50% (9/18) of participants with smart watches logged physical activity data. Significant differences in psychosocial profile were observed at baseline between the groups. The Child Behavior Checklist (CBCL) mean total score was 71.7 (SD 3.1) in the intervention group vs 57.6 (SD 6.6) in the control group, *t*-test *P*<.001, and also different among those who completed the planned protocol compared with those who withdrew early (CBCL mean total score 59.0, SD 9.3, vs 67.9, SD 5.6, respectively; *t*-test *P*=.04).

**Conclusions:**

A high early attrition rate was a key barrier to full study implementation. Perceived task burden in combination with behavioral issues may have contributed to attrition. Low exposure to the experimental intervention was explained by poor acceptability of Mandolean as a home-based tool for treatment. Self-monitoring using myBigO and the smartwatch was acceptable among this cohort. Further technical and usability studies are needed to improve adherence in our patient group in the tertiary setting.

## Introduction

### Background

Global prevalence rates of childhood obesity were estimated at 7.8% for boys and 5.6% for girls in 2016, and prevalence is increasing in low-income countries and communities [1]. Interventions in childhood are critical, as children with obesity experience a range of physical and psychosocial health issues and are at a high risk of developing chronic disease in adulthood [2]. Diet, physical activity, and other behavioral interventions can be effective in terms of change in adiposity, and significant clinically relevant metabolic benefits have been demonstrated with a 0.25 reduction in BMI z-score [3], although meaningful reductions in cardiometabolic markers are observed with reductions of 0.15 [4,5]. Recent Cochrane meta-analyses of behavior change interventions reported 12-month reductions in BMI z-score of −0.06 units among children aged 6 to 11 years [6] and −0.13 units (95% CI −0.21 to −0.05) in adolescents aged 12 to 17 years [7].

There is growing evidence that eating behaviors, not simply driven by food choice, influence energy consumption, appetite, and satiety. Fast eating is associated with high body weight [8], and interventions to reduce the eating rate seem to enhance weight loss [9]. Eating food quickly may contribute to blunted responses to normal satiety signals, whereby an individual does not respond to gastric distension and gut peptide release, so that normal appetite suppression pathways do not function as expected during fast eating occasions [10,11]. A reduction in the eating rate aimed at reducing portion size and normalizing satiety signaling has been recently studied [10-16]. A study of adolescents aged 9 to 17 years found that an eating rate intervention enhanced weight loss at 12 months compared with usual care (change in BMI z-score of −0.27) [16]. Slowing eating rate can also reduce self-selected portion size with no reduction in postmeal satiety levels among children and teenagers [10,13,16]. A recent review appraised a number of commercial apps targeting appetite regulation [17]. Research-driven interventions include real-time technology-assisted tools for meal times, including utensils with vibrotactile feedback [12,14] and Mandolean, a plate scale measuring eating rate with real-time computer or smartphone feedback [13,15,16]. Mandolean has shown promise for the treatment of childhood obesity [16].

Physical activity in combination with dietary behavior change, rather than either in isolation, is the recommended component of interventions for childhood obesity [6,7,18]. The use of wearable accelerometers to measure physical activity is the accepted objective means of measurement in free-living individuals [19], which can be used to determine energy expenditure and requirements [20], and the time spent in high-intensity physical activity determines variation in childhood cardiometabolic risk factors [21]. One of the advantages of mobile health (mHealth) interventions compared with traditional approaches is that data from monitoring tools and participant engagement are provided objectively.

Despite improved technologies and access to mHealth tools for the purpose of monitoring health status and implementing interventions for health behavior change [22,23], challenges with adherence and exposure remain [24,25]. Planned exposure, impact, and potential outcomes are altered by participants’ interaction with study tools and technology [13,15,24]. The importance of content, design, and testing periods with the target group has been emphasized as a means of enhancing engagement with mHealth apps [24,25]. Reporting process measures is increasingly important, as they contribute to moving the field forward and providing translational accuracy in research and practice [26].

Interventions that improve and expand treatment options for children with obesity are important because of challenges within traditional clinical care, including available time and resources that impact access for service users. mHealth tools provide adjunctive options to standard treatment approaches and can be beneficial for patients at home and their clinical team. However, engagement with devices and apps can act as a barrier to treatment [13,15,22].

### Objective

The aim of this study was to determine the feasibility and acceptability of an intervention using 2 mHealth apps among children in the treatment for obesity in a tertiary outpatient setting. As diet and physical activity interventions are typically undertaken together, it was of interest to assess the acceptability of Mandolean in addition to a physical activity monitoring tool.

## Methods

### Study Design

This study was conducted to determine the feasibility and acceptability of a proposed mHealth intervention. The study was not registered as a trial; however, a randomized design was implemented to ascertain protocol feasibility for a proposed randomized controlled trial. We evaluated the process of using 2 mHealth smartphone apps among children and adolescents receiving treatment for obesity. Feasibility measures included recruitment rates and procedures, and retention rates. Fidelity encompassed intervention delivery and adherence to randomization and study procedures. Retrospective acceptability included objective measures (engagement with smartphone apps) and self-reported measures (system usability score surveys and verbal feedback).

### Participants

Children and adolescents (aged 9-16 years), with a diagnosis of clinical obesity (BMI >98th percentile for age and sex), referred to the W82GO Child and Adolescent Obesity Service at Children’s Health Ireland at Temple Street, Dublin, Ireland, were eligible to participate. Socioeconomic status was indicated by the Pobal HP Deprivation Index for Small Areas [27]. Children and adolescents were required to have access to a smartphone (phones with Android operating system version 6.0 or above were compatible with the smartwatch and myBigO at version 0 when used in the feasibility study and both Android and iOS were compatible with Mandolean, which did not undergo further development during the study). Smartphone literacy was assumed, and training was provided on study apps by a researcher at baseline who established competency in study tasks. Exclusion criteria included moderate or severe learning difficulties that would prevent the use of smartphone apps or giving informed assent, the child having a concurrent serious medical issue, if the parent or child was not proficient in understanding English, refusal by the child to give assent or parents/legal guardians to give informed consent to participate in the project, or if the child lived in Direct Provision (the system of asylum seeker accommodation used in the Republic of Ireland). Pregnancy and the use of medications known to affect weight also precluded participation.

### Study Procedures

Health professionals working within the obesity service informed eligible participants about the study and provided a patient information leaflet to parent(s)/legal guardian(s) and their child. After 3 to 7 days, a researcher contacted the parent/guardian by phone to answer questions and check whether they wished to participate. Once written informed consent and child assent were received, a study appointment was offered for baseline assessment. A single dietetic researcher (SB) implemented the study protocol, coordinating, and providing communication. All participants met a researcher for scheduled face-to-face and phone communication and were also invited to call or email the researcher outside scheduled reviews. Study contacts at each time point (T), modality, and actions/measures for each contact are detailed later. At baseline, the researcher guided participants through a practice meal of their choice (brought to the hospital by participants) using Mandolean (Multimedia Appendix 1 shows a more detailed protocol). When participants completed a meal using Mandolean, data were available to the research team via a dedicated web-based clinical portal (Mandobase).

At present, the Big Data Against Childhood Obesity (BigO) project is testing the myBigO app and clinical portal [28]. The app aims to gather behavioral data alongside measures of environmental conditions (eg, urban built environment, infrastructure for physical activity, food marketing) among young people in general and an age-matched clinical cohort with obesity. Aggregation mechanisms are being developed to correlate population behaviors with environmental characteristics for the purpose of highlighting priority public health interventions [29].

All participants registered with the myBigO app and were set up with their smartwatch at baseline and postintervention. The default method of data synchronizing between the smartwatch and myBigO on the smartphone was a wireless internet connection (to avoid potential expense by using participants’ personal mobile data). To ensure that the BigO system received regular accelerometer data, participants and their parent(s) were shown how to check the Bluetooth and internet connection between the smartwatch and phone, and they were asked to repeat this process every evening. For consistency, standard verbal, practical, and written instructions to take home were provided to participants and their caregivers (Multimedia Appendix 2). When participants completed a meal using Mandolean and wore the smartwatch, the data were available to the research team via web-based clinical portals (Mandobase and BigO clinical portal; see Multimedia Appendix 3 for detail of baseline and postintervention measures).

#### Usual Clinical Care

**Figure 1:**
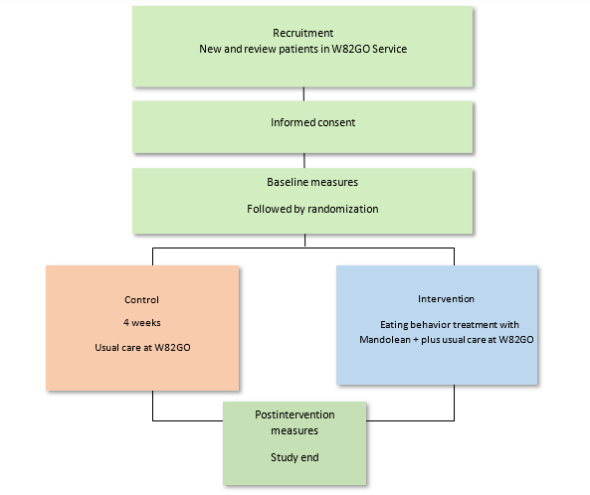
Flowchart of study protocol for a mobile health randomized feasibility study for an eating behavior intervention with children (aged 9.0-16.9 years) in the treatment of obesity in a tertiary health care setting. Baseline and postintervention measures include anthropometry, questionnaires (Child Behavior Checklist, Pediatric Quality of Life, Piers-Harris, Dutch Eating Behavior Questionnaire, and, at study end, evaluation questionnaires and System Usability Scale [31]), rate of eating using Mandolean, and physical activity levels with the smartwatch and myBigO app.

The W82GO Child and Adolescent Weight Management Service is a multidisciplinary obesity service that delivers efficacious obesity interventions [30]. Children and adolescents aged ≤16 years with a BMI >98th percentile are referred to the service by hospital physicians based at Children’s Health Ireland at Temple Street. On referral, children and their caregivers are invited to a multidisciplinary clinic and undergo assessment by a pediatric dietitian, a pediatric physiotherapist, and a pediatric psychologist. On the basis of the needs of the child and family, a treatment plan is developed, and patients are offered either group-based treatment or treatment delivered in a 1:1, more traditional outpatient setting. Treatment is family based and was developed using contemporary scientific evidence [30]. Participants allocated to the usual care arm completed baseline testing, followed by 4 weeks of usual clinical care and subsequent retesting (Figure 1).

#### Intervention

Mandolean was developed by the Section of Applied Neuroendocrinology, Karolinska Institute, and Mando Group AB, Stockholm, Sweden. It consists of a plate scale that is wirelessly connected to a smartphone app with 2 main functions: (1) measures the rate of eating and (2) provides the user with visual feedback on slowing the rate of eating. The intervention arm involved usual care with additional training to reduce the rate of eating using Mandolean. Following randomization, the participants assigned to the intervention group received additional instruction on using the training functions of Mandolean for at least one meal per day (lunch, dinner, or both) over 4 weeks (minimum planned dose exposure: 28 meals). Using Mandolean training functions, the patient learns to adopt a typical pattern of eating and satiety by following the displayed *ideal* rate of eating, which they aim to match. The clinician used baseline data (usual portions sizes and rate of eating) to guide a *training meal* program for the user. The training aims to teach the patient to eat 280 to 350 g in 13 to 15 min and to perceive a level of satiety of *moderately full* by the end of the meal. A full description of the Mandolean training procedures is included in Multimedia Appendix 1. The use of Mandolean in this study integrated a number of behavior change components, categorized according to the behavior change technique taxonomy by Michie et al [32], including goals and planning, feedback and monitoring, social support, shaping knowledge, comparison of behavior, repetition and substitutions, and antecedents (Multimedia Appendix 4 gives the subtechniques used).

### Sample Size

A sample size of 20 participants was the target, which was considered sufficient to evaluate the process measures in this randomized feasibility study and is in line with similar studies [10,13].

### Randomization

Using a web-based randomization service (Sealed Envelope), participants were randomized to Mandolean eating behavior training intervention or control group (Figure 1) by 1 researcher (SB). Age (9-11.9 years and 12-16.0 years) and gender stratifications were applied, and participants’ parents were informed of their treatment group by phone after baseline measurements. Participants, therefore, were aware of their study allocation, as the intervention required exposure to new eating behavior training. At this point, further study review appointments were planned which are outlined in the results section.

### Outcomes

The Consolidated Standards of Reporting Trials (CONSORT) extension for randomized feasibility studies was used to guide transparency and quality in reporting study measures (Multimedia Appendix 5) [33,34]. Trial process–related outcomes measured in this study addressed (1) feasibility (recruitment process, rates of recruitment to the study, and rates of retention and attrition of the study arms), (2) fidelity (adherence to randomization protocol, appointment attendance [number, modality, and duration of study appointments], dose delivered [study tasks planned and completed], dose received [training exposure logged in Mandolean clinical portal], and adherence to intervention procedures), and (3) acceptability (participant engagement with the Mandolean app [number of training meals completed], participants’ engagement with the BigO physical activity monitoring app [volume of data collected], and scores from the system usability scale [SUS] questionnaires [31]).

### Data Analysis

Statistical methods for quantitative measures included descriptive frequencies and standard *t* test comparisons between intervention and control groups and completer and noncompleter groups for baseline age, anthropometry, and questionnaire scores. Qualitative feedback from questionnaires and discussions with users was analyzed for content and categories created for the purpose of presenting key acceptability issues, challenges, and facilitators for users and health care professionals.

### Ethics

The research protocol was reviewed by the research ethics committee at Temple St. Children’s University Hospital and approved on August 08, 2018 (number 18.013). A pseudonymized patient identification coding system was incorporated and stored in an encrypted file at the clinical site, so that no personal patient information was shared or processed via mHealth apps. Data collected on the apps were locally transformed on the participants’ mobile phones, and the transformed data, not containing identifiable information, were transmitted and stored to the respective clinical portals (for Mandolean and BigO).

## Results

### Participants

Participants were recruited between May 2018 and February 2019. Table 1 describes participants’ characteristics and baseline assessment measures for the intervention and control groups. A total of 63% (5/8) of participants in the intervention group and 42% (5/12) of participants in the control group were categorized as being below average socioeconomic status (Multimedia Appendix 6 gives further detail). No significant differences between the intervention and control groups were noted for mean age, BMI, or BMI SD score (SDS). Differences in mean total score, externalizing behavior total score, and internalizing behavior total score for the parent-reported Child Behavior Checklist (CBCL) were observed (Table 1). A significantly higher score in mean baseline total T score for CBCL was observed between those who completed the study and those who did not, indicating more behavioral issues among those who withdrew. Owing to the high attrition rate, completer and noncompleter groups are also presented in Table 1.

**Table 1:** Characteristics of participants in a randomized feasibility study for a mobile health eating behavior training intervention at baseline and follow-up.

Participants’ characteristics and baseline measures			Intervention (n=8)	Control (n=12)	Completed the study (n=12)	Did not complete the study (n=8)
**Sex**						
	Male, n (%)		3 (37)	6 (50)	4 (33)	5 (63)
	Female, n (%)		5 (63)	6 (50)	8 (67)	3 (37)
Age (years), mean (SD)			13.1 (2.3)	13.5 (2.3)	13.3 (2.7)	13.5 (1.5)
Baseline BMI (kg/m^2^), mean (SD)			31.6 (3.9)	33.2 (5.9)	32.16 (5.7)	33.1 (4.6)
Baseline BMI SD score, mean (SD)			3.02 (0.27)	3.04 (0.60)	3.00 (0.56)	3.09 (0.37)
Stage of usual care (weeks), mean (SD)			40.1 (46.2)	17.7 (16.8)	26.9 (33.1)	26.3 (34.6)
**Baseline physical and psychosocial health self-report**						
	**Child or adolescent self-report questionnaire score, mean (SD)**					
		Physical health PedsQL^a^ [35]	74.6 (17.1)	69.1 (15.1)	70.5 (17.6)	72.3 (13.3)
		Psychosocial health PedsQL	49.0 (24.87)	64.7 (20.5)	63.3 (19.5)	51.2 (27.5)
		DEBQ^b^ external eating [36]	1.58 (0.72)	2.00 (0.62)	2.00 (0.56)	1.58 (0.79)
		DEBQ emotional eating score	1.29 (0.76)	1.50 (0.53)	1.63 (0.61)	1.09 (0.52)
		DEBQ restrained eating score	1.91 (0.81)	2.03 (0.30)	2.08 (0.30)	1.84 (0.79)
	**Parent self-report questionnaire score, mean (SD)**					
		CBCL^c,d,e^ total T score [37]	71.7 (3.1)	57.6 (6.6)	59.0 (9.3)	67.9 (5.6)
		CBCL externalizing behavior T score^f^	67.8 (4.7)	57.2 (7.8)	58.2 (7.5)	65.0 (8.7)
		CBCL internalizing behavior T score^g^	64.3 (6.2)	53.8 (8.5)	56.1 (9.5)	60.3 (9.2)

^a^PedsQL: pediatric quality of life.

^b^DEBQ: Dutch eating behavior questionnaire.

^c^CBCL: Child Behavior Checklist.

^d^CBCL total T score. Intervention group versus control group: *t* test for equality of means, equal variances assumed *P*<.001.

^e^CBCL total T score. Completed the study group versus did not complete group: *t* test for equality of means, equal variances assumed *P*<.04.

^f^CBCL externalizing T score. Intervention group versus control group: *t* test for equality of means, equal variances assumed *P*=.02.

^g^CBCL internalizing behaviour T score. Intervention group versus control group: *t* test for equality of means, equal variances assumed *P*=.01.

### Feasibility: Rate of Recruitment

**Figure 2:**
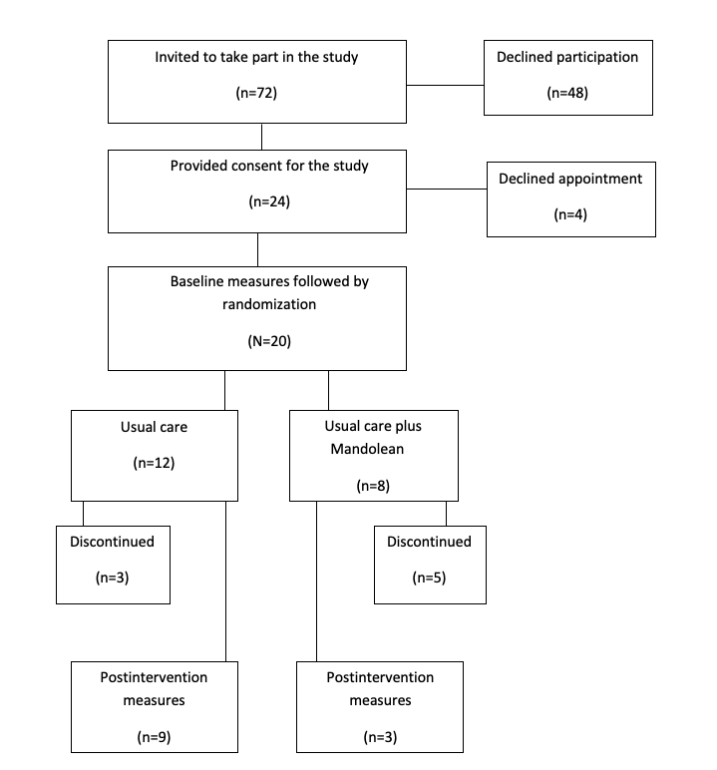
Consolidated standards of reporting trials diagram for pilot randomized trials.

Children and adolescents were recruited between June 2018 and January 2019. One strategy of recruitment, which involved offering patients and their families study recruitment packs at their first multidisciplinary assessment appointment for the obesity service, was discontinued during the feasibility study. Families reported mixing up the recruitment pack with usual care information received on the same day and had not read the study information by the time the researcher contacted them some days later by phone. Instead, a researcher or clinician provided a 5-min information session and study information pack to parents and their children when they were established within the service and followed up with a phone call 3 to 7 days later. In total, 72 eligible parent-child dyads took recruitment packs for the study, and 33% (24/72) signed informed consent to participate. Following this 28% (20/72) attended for the first study appointment (Figure 2 shows participant flow through the study).

### Feasibility: Retention and Attrition

A total of 25% (3/8) of participants in the control arm and 63% (5/12) of participants in the intervention arm withdrew early. Characteristics, participation levels, and feedback from children and adolescents who dropped out of the feasibility study before completion are presented in more detail in Multimedia Appendix 7.

### Fidelity: Adherence to the Randomization Protocol

The web-based randomization process resulted in males being underrepresented in the intervention group.

### Fidelity: Appointment Attendance

Intervention participants attended study appointments in addition to usual care (Table 2). There was good adherence to planned face-to-face appointments at time point 1 (T1); however, there was mixed adherence thereafter, with the exception of phone reviews provided by the researcher. The time allocated to the study appointments was appropriate. Illness, school and family commitments, competing appointments at the hospital, and living a long distance from hospital (as perceived by families) were barriers to attending appointments. Final reviews were completed by phone for 3 families who lived at a distance to the hospital to minimize absenteeism from school/work.

**Table 2:** Fidelity with planned actions and measures at each time point.

Actions and measure		Time point					
		T1 (week 0)	T1a (T1+1 week)	T2 (T1+2 weeks)	T2a (T1+4 weeks)	T3 (T1+6 weeks)	T4 (T1+8 weeks)
Planned mode		In person	Phone	Phone or in person	Phone	In person	In person
Mode adherence^a^, n (%)		20 (100)	16 (80)	17 (85)	12 (60)	8 (40)	9 (45)
Time allocated, min		60	15	30	15	30	30
Time allocated adherence^a^, n (% of those who attended)		17 (85)	16 (100)	16 (94)	12 (100)	7 (88)	9 (100)
Smartwatch and myBigO setup^a^, n (%)		12 (60)	5 (25)	1 (5)	N/A^b^	6 (30)	N/A
Mandolean app installation and baseline meal demonstration^a^, n (%)		16 (80)	N/A	N/A	N/A	N/A	N/A
Mandolean intervention training meal demonstration (n=8), n (%)		N/A	N/A	4 (50)	N/A	N/A	N/A
Intervention verbal instructions (n=8), n (%)		N/A	N/A	5 (63)	N/A	N/A	N/A
Intervention standard instructions (n=8), n (%)		N/A	N/A	5 (63)	N/A	N/A	N/A
Anthropometry^a^, n (%)		15 (75)	N/A	N/A	N/A	N/A	9 (45)
Sociodemographic data^a^, n (%)		20 (100)	N/A	N/A	N/A	N/A	N/A
**Questionnaires, n (%)**							
	Dutch eating behavior	19 (95)	N/A	N/A	N/A	N/A	N/A
	Piers-Harris	19 (95)	N/A	N/A	N/A	N/A	N/A
	Child behavior checklist parental questionnaire	19 (95)	N/A	N/A	N/A	N/A	N/A
	Pediatric quality of life	19 (95)	N/A	N/A	N/A	N/A	N/A
Mobile health app usability questionnaires, n (%)		N/A	N/A	N/A	N/A	N/A	11 (55)

^a^Adherence to planned protocol expressed as % of 20 participants recruited to study at baseline unless otherwise indicated.

^b^N/A: not applicable at given time-point.

### Fidelity: Dose Delivered and Adherence to Intervention Procedures

Table 2 shows adherence to intervention protocols at each time point. The reasons for an incomplete smartwatch setup at T1 included an incompatible smartphone (n=5), the parent could not remember personal account password to complete syncing with the app (n=2), and insufficient time (n=1). In total, 5 patients took written instructions for smartphone installation at T1 and completed the process at home on a compatible smartphone belonging to another parent or sibling. Two patients did not complete the Mandolean setup at T1 because of patient time constraints, and this was planned for time point 2 (T2), which was completed with 1 participant, and the other did not attend T2 or subsequent appointments. Two patients did not complete questionnaires at baseline, because of lack of time, and were asked to return by post or at the next appointment, of which 1 set was returned.

In terms of intervention implementation, of 8 patients randomized to Mandolean treatment, 4 received demonstration and instructions by the researcher in person; 1 patient was provided with instructions over the phone and written instructions by post, and 3 patients did not attend T2 to commence training (Table 2).

### Fidelity: Dose Received

#### Smartwatch and myBigO Apps

An early version of the myBigO app used here (which accessed accelerometer data via smartwatches) was compatible with Android operating systems 6.0 and above. Of the 18 children set up with smartwatches at baseline, 11 were connected to parents’ phones, 1 was connected with a sister’s phone, and 6 children and adolescents used their own phones. Available data from the BigO system indicated that of the 18 smartwatch setups with myBigO, 50% (9/18) of participants contributed some data. Of those who contributed data, the range was highly variable from 0.3 to 9.2 days (mean 1.6, SD 2.9 days; median 0.2; IQR 2.0). Two participants did not wear the smartwatch at all after the baseline setup (one because of illness and the other was self-conscious about wearing at school) and subsequently dropped out. Two parents deleted the myBigO app at some point during the study (one because of lack of space on the phone and the other because the father thought the child was no longer using the watch/app). One child did not live with the parent who had myBigO and the smartwatch setup with a compatible smartphone and, as a result, did not synchronize regularly. Attrition (n=8), poor attendance at time point 3 (n=2), technical challenges resynchronizing watch to phone (n=3), and strap breaking (n=1) were reasons for low usage postintervention. In addition, self-reported days wearing the watch among users at the postintervention stage was highly variable. Two patients reported sensory issues and disliked wearing the watch. A short battery life and forgetting to charge or wear were commonly reported by patients for not wearing as advised.

#### Mandolean

All participants completed at least one Mandolean baseline meal that measured their rate of eating. Three participants completed 1 baseline meal with the researcher at the hospital canteen and did not complete any at home. The remaining participants successfully completed some baseline measurement meals at home, with a range of usage (2-19 meals). Participants’ engagement with Mandolean and exposure to the planned intervention are presented in Table 3.

**Table 3:** Summary statistics for the use of Mandolean device at baseline (all participants), intervention phase (intervention only), and postintervention (all participants).

Variable	Baseline meal frequency	Training meals frequency	Postintervention meals frequency
Number children/teenagers, n (%)	20 (100)	8 (40)	12 (60)
Mean (SD)	5.7 (5.2)	5.4 (9.2)	4.6 (5.2)
Median	5.0	1.0	3.5
25th centiles	1.3	0.0	0.5
75th centiles	9.0	9.3	7.8
Mean meals as percentage of planned meals	56	19	46
Unsuccessful meal attempts^a^, mean (SD)	1.2 (2.1)	0.5 (0.9)	1.0 (0.8)

^a^Unsuccessful meal attempts were meals initiated and therefore registered on the clinical portal system, but ultimately did not record the rate of eating successfully.

Of the 8 participants allocated to the intervention training, 5 received training instructions and 3 did not engage with the training component. One participant subsequently dropped out of the study before training commenced; therefore, the final number of participants exposed to intervention training meals was 50% (4/8 of those randomized to training). Exposure at an individual level represented 7% (2/28 planned meals; male aged 12.3 years), 14% (4/28 planned meals; female aged 9.5 years), 39% (11/28 planned meals; female aged 15.2 years), and 93% (26/28 planned meals; female aged 11.6 years) of planned intervention.

### Acceptability

#### Participants’ Engagement With Mandolean and the BigO Physical Activity Monitoring App

The findings relating to the dose received presented earlier indicated poor acceptability as measured through active engagement among users.

#### Self-Report Acceptability Measure: System Usability Scale

**Figure 3:**
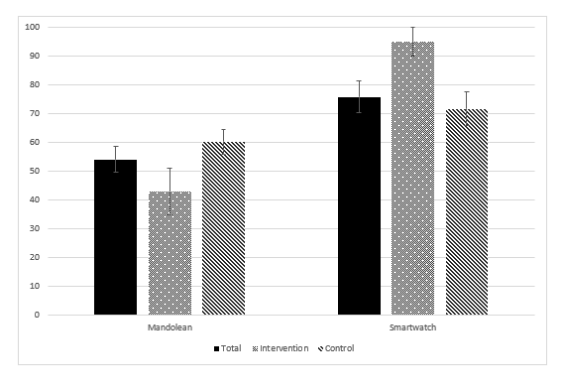
Bar chart showing participants’ postintervention System Usability Scale scores for Mandolean and smartwatch for the total (n=11), intervention (n=4), and control (n=7) groups.

The mean SUS score results are illustrated in Figure 3. A score of 68 or greater is considered acceptable when assessing the user experience of technology [31]. Mandolean did not achieve a mean greater than 68 for the total group or within any subgroup, and the smartwatch achieved a mean of 68 or greater for all groups. A more detailed table of results is presented in Multimedia Appendix 8.

### Acceptability: Qualitative Feedback From Participants and Their Parents

Qualitative feedback from participants and their parents was categorized for each piece of technology. The main barriers to using Mandolean were (1) connectivity issues; (2) difficult, awkward, or time consuming to set up, which interfered with family meal times; (3) incompatible with family routine (ie, no regular family meal times, summer holidays, or parental shift work); and (4) forgetting to use. A small number of children and parents reported becoming more aware of their speed of eating as a result of using Mandolean. Most participants enjoyed wearing the watch, liked the timekeeping function, and self-monitored their daily activity levels. The main drawbacks noted were (1) a short battery life, (2) sensory issues and finding the watch uncomfortable, and (3) feeling self-conscious at school. Participants’ quotes and more details are presented in Multimedia Appendix 8.

## Discussion

### Principal Findings

We conducted a study to determine the feasibility and acceptability of a proposed intervention using 2 mHealth apps among children and adolescents being treated for obesity. The study process was documented thoroughly, and based on the observed results, we concluded the need for further technical usability testing in this population. A slow recruitment rate, high attrition rate, and low fidelity with planned interventions were the key outcomes informing feasibility. Greater psychosocial issues among the intervention and noncompleter groups, observed in the baseline behavioral questionnaire (CBCL), were also noteworthy in this cohort. Although we cannot imply causality about the effect on study engagement or attrition, this finding provides important contextual background about individual and group characteristics, which may have contributed to suboptimal usage of mHealth tools at home.

In terms of delivering study components, we found realistic time points and modes of delivery. Poor fidelity with participants’ exposure to intervention components, in particular, the low number of participants randomized to the intervention who attended the necessary training, and the low level of engagement with training meals on Mandolean were the primary barriers to intervention implementation. Adherence with the smartwatch set up at baseline was for the majority achieved, whereas fidelity postintervention was problematic because of attrition and nonattendance for reviews.

We considered the high attrition rate as a signal of poor acceptability of the intervention, particularly when a greater number of intervention arm participants opted to leave the study before completion. The poor rating of Mandolean on the SUS scores provided further evidence. In contrast, positive feedback about using and understanding the smartwatch and the myBigO app was received. Despite the acceptability of the smartwatch, the wide range of exposure levels is suggestive of underlying barriers that need to be understood if we are to maximize adherence to mHealth adjuncts to clinical care. Sekhon et al [38] proposed acceptability as multifaceted to include the following 7 component constructs: affective attitude, burden, perceived effectiveness, ethicality, intervention coherence, opportunity costs, and self-efficacy. Applying these constructs to our SUS and qualitative findings, we suggest that technical difficulties, perceived awkwardness, and time cost associated with using Mandolean contributed to a negative attitude among participants and their parent(s). These, in turn, possibly contributed to feelings of high burden and low confidence in completing the required study tasks. There is also the possibility that the perceived burden associated with Mandolean affected overall study task adherence, including the smartwatch and myBigO.

### Comparison With Prior Work

Our study with Mandolean differs from previously published work in 2 technical ways: (1) we used a new mobile tool, with a smartphone app interface for Mandolean, whereas others plugged it into a computer [15,16], and (2) we had access to objective engagement data from a clinical portal facility that was not available for an earlier clinical study [16]. Compared with a community-based feasibility study that reported process measures, we had an older cohort (9-16.0 years vs 6-11.0 years), we recruited in the hospital setting, and we also incorporated an additional mHealth tool to measure physical activity levels [15]. Intervention exposure in a more recent study using smartphones mirrored our findings that engagement with Mandolean meals at home among adolescents varied considerably within the intervention group from 5 to 80 meals (median 28 meals), out of a planned 1 meal per day for 6 months [13]. A study of younger children found that just 19% achieved the minimum expected usage of 5 meals per week with Mandolean [15]. These studies and others [39] reported engagement issues among children and adolescents when mHealth apps are considered burdensome.

Individual factors contributing to poor adherence with wearing the smartwatch that we found, including early attrition, sensory issues, forgetting to charge, forgetting to wear, and feeling self-conscious, are similar to other studies using wearable devices with young people. Although rigorous research with smartwatches as activity trackers is in its infancy in comparison with traditional accelerometers [40], some of the same adherence issues may apply. For example, Jago et al [41] reported considerable variability in adhering to a 7-day accelerometer among children, finding that parents would forget to put on the accelerometer, some children found it uncomfortable, and some were self-conscious about wearing it at school; however, others were excited or interested in wearing it. We found similar barriers among our older group and facilitators among younger participants. Research with a commercial fitness tracker among adolescents with obesity reported a discontinuation rate of 68% before the end of the study that was linked to barriers to physical activity not being addressed by a tracker, seasonality, feelings of activity incompetence, and gradual withdrawal of parental and clinical support [42]. Our finding that high acceptability of a smartwatch does not translate to adherence is also reported by Phan et al [42]. The physical activity self-monitoring tool available on the smartwatch screen in our study was cited by a number of children and parents as a benefit because they were trying to increase their activity levels. Formal integration of recognized and important behavioral change techniques for *self-monitoring* and *goal setting* [32] could be maximized for future studies with research-led apps such as myBigO and smartwatches. Retention in clinical research, however, is challenging, and we note that a two-fold study initiation rate may be required to achieve meaningful physical activity data for clinicians treating children in treatment for obesity.

Although withdrawal of 63.5% of participants in the intervention arm here is considerably higher than that in previous studies using Mandolean [13,15,16], the psychosocial profile of participants has not been described by others. Therefore, we cannot assume that participants were similar across published trials. The most comparable health care setting and age group to our own is the study by Ford et al [16]. They demonstrated high compliance with the intervention, particularly among the Mandolean arm, with 83% of participants attending all study appointments. High-intensity contact (14 appointments in 12 months) was reported as a facilitator of retention [16]. Meta-analyses in the wider electronic health (eHealth) literature show attrition rates of 12% to 29% [43]. Attrition can depend on a range of factors known to be influential in clinical care (eg, school absenteeism, dissatisfaction with care components, demographic factors) [44-46], in addition to factors influencing dropout from eHealth-specific interventions (eg, registration requirements and male gender) [47-49]. One unique aspect of this study was the combination of 2 mHealth tools for measuring both dietary behavior and physical activity. Our attrition rate, therefore, may not be informative for all mHealth interventions with children and adolescents but certainly contributes insight where high participant engagement is expected among groups with complex health and psychological needs.

The predominant technical issues identified were difficulty connecting scale to phone, Mandolean failing to recognize the plate when weighing out food, and loss of connection midmeal, which others also report as a barrier to compliance at home [13,15]. User experiences such as *annoying*, *cumbersome*, and *time consuming* reported by Hamilton-Shield et al [15] reflected the feedback from our participants, despite using an updated mobile app version of Mandolean. We also found a number of other practical challenges similar to Hinton et al [13], including child impatience during setup, interruption of the flow of family mealtimes, difficulty with the portion size limit for training meals, and forgetting to use. A minority of parents in this study reported more positively that children adapted quickly to the routine of using Mandolean at mealtimes, particularly younger participants. In contrast, parents whose teenagers withdrew from the study early would have preferred them to complete the study. In a community study, by 4 to 10 weeks, some children lost interest, and a few parents were tired of using the Mandolean, particularly setting up, dishing food onto a plate, and weighing food [15]. Although their trial was a longer duration, we found that these reactions were evident earlier (from 2 weeks onward) and, in some cases, contributed to early withdrawal from the study.

Higher behavioral problems among the intervention group at baseline appeared to be a random outcome of allocation. The subsequent high attrition within the group suggests that psychosocial issues in combination with the intervention burden discussed earlier may have played a role in early attrition. The reasons for attrition among children with obesity vary in the intervention literature. Some interventions report no differences between completers and noncompleters for behavior measures using CBCL [50,51]. Others have shown that the social competence of children aged 8 to 14 years who are overweight or obese, defined using the CBCL, was one of a number of predictors (including lower baseline weight and Caucasian parents) of BMI SDS reduction following a 12-month intervention [45]. Behavioral problems in children from disadvantaged areas have been linked to adherence in other conditions such as type 1 diabetes [51]. Behavioral problems are associated with a high risk of overweight and obesity among children, independent of other risk factors such as parental obesity, education, poverty, and race [52,53]; therefore, we expected to find behavioral issues among a substantial number of our participants. Participants here may not be representative of the general population of children and adolescents with obesity for a number of potential reasons, including obesity severity that prompted a referral to the specialist pediatric service, varying motivation depending on the stage of treatment, and interest in joining research studies based on health status. Although patients with known behavioral disorders were ineligible for participation in this study, a self-report tool at baseline assessing psychosocial and behavioral issues detected underlying behavioral issues that may have affected children’s ability to partake fully in research tasks (and hence treatment tasks). On the basis of this experience, we recommend multidisciplinary baseline assessments to include behavioral measures for similar adjunct interventions with children receiving treatment for obesity.

This is the first published study in which myBigO with a smartwatch was incorporated into an intervention in a clinical setting. The process outcomes provide some lessons for future research and practice. To fully describe the feasibility phase process, we included all participants who attended a baseline appointment. The slow uptake and early attrition observed indicates that children in treatment for obesity may require greater choice or flexibility in how they contribute data and benefit personally from participation. Different approaches to recruitment and deployment need to be explored with different subgroups, including those with psychosocial or behavioral issues and different age groups (children vs early adolescents vs midadolescents), as they have varied motivations. Greater self-monitoring functionalities and reminders to charge and wear smartwatches may also improve adherence based on barriers reported. Given our challenges with a postintervention accelerometer period, one unbroken smartwatch exposure may be more realistic and preferable among children in treatment for obesity, reducing the need for extra face-to-face review appointments. However, clinicians wishing to observe behavior pre- and postintervention may wish to examine the feasibility of a longer intervention period in which participants would have time to implement and monitor goals based on baseline measures.

### Limitations

This is a feasibility study with a small sample size and short intervention period. Recruitment was limited to children and adolescents attending 1 specialist pediatric obesity program, which may have limited recruitment rate and introduced population bias in terms of obesity severity and associated complications and participant motivation to participate in the research. The recruitment rate was also delayed at the study outset by the implementation of the General Data Protection Regulation in the European Union and Irish interpretation of the regulation for the purposes of health research. Poor adherence to treatment was evident at early stages; therefore, a longer intervention with Mandolean was unlikely to add additional benefit or knowledge in this group. The intervention group received additional training on Mandolean that the control group was not exposed to which heightened awareness about behaviors of interest and combined with task burden could well introduce unknown biases. A more structured and validated technical usability study may be of benefit to further understand the challenges children and their families face in using the mobile Mandolean system. Although the SUS has been used previously to evaluate adult user experience with wearable devices [54], the survey has not, to our knowledge, been validated in a pediatric population. However, it has advantages for use with children, as it is short and uncomplicated. It is suitable for use with small samples, provides space for comments, and a final score can be interpreted with an established reference standard. The smartwatch with myBigO was in the first stage of feasibility testing in this cohort, and, as such, the technology will continue to be developed based on users’ needs. The attrition and engagement measures should be interpreted in the context of a feasibility study with 2 mHealth apps aimed at children with obesity and, as such, not indicative of the performance of either tool used in isolation.

### Conclusions

Our study explored the process outcomes of using mHealth tools in an obesity treatment study and highlighted challenges and opportunities related to feasibility, fidelity, and acceptability. By transparently reporting feasibility using the CONSORT extension guidance [33], we reported potential challenges for mHealth interventions among children with obesity. mHealth interventions are rapidly progressing; however, we need to be cautious in terms of efficacy, burden to participants, and our responsibility to identify vulnerable subgroups at baseline. Challenges include task burden and adherence to complexity in mHealth systems recommended for use at home, particularly among families experiencing behavioral issues. The opportunities noted here include high acceptability of a self-monitoring physical activity system where data are shared between patients and healthcare workers. Children with obesity attending treatment have complex needs and, given health service limitations, would benefit from adjuncts to traditional treatment that can be implemented outside of clinical settings. Additional technical and usability studies are recommended to improve our understanding of adherence to treatment.

## Appendix

Multimedia Appendix 1 Protocol and process for training participants to use Mandolean.

Multimedia Appendix 2 Written standard instructions provided to the participants.

Multimedia Appendix 3 Description of baseline measurements.

Multimedia Appendix 4 Behaviour Change Techniques incorporated into the intervention.

Multimedia Appendix 5 Consolidated standards of reporting trials of electronic and mobile health apps and online telehealth checklist report.

Multimedia Appendix 6 Bar chart showing participants’ socioeconomic position.

Multimedia Appendix 7 Characteristics and feedback from children and teenagers who dropped out before completion.

Multimedia Appendix 8 System usability scores for Mandolean and a smartwatch, including qualitative comments.

## Abbreviations

BigO: big data against childhood obesity
CBCL: Child Behavior Checklist
CONSORT: consolidated standards of reporting trials
eHealth: electronic health
mHealth: mobile health
SDS: SD score
SUS: system usability scale
