# Supplementary material for: Mobile Health Apps in Pediatric Obesity Treatment: Process Outcomes From a Feasibility Study of a Multicomponent Intervention
Source: JMIR Mhealth Uhealth. 2020 Jul 8;8(7):e16925. doi: 10.2196/16925 (PMC7381070; doi:10.2196/16925)
Supplement: Multimedia Appendix 1 [file mhealth_v8i7e16925_app1.docx]

1. **Mandolean®:** **Protocol and process for training participants to use Mandolean®**
   1. Prior to training session, the researcher creates unique login credentials for the participant which is linked to a pre-assigned plate scale.
   2. Participants were asked to bring a meal of their choice, that was suitable to eat with knife and fork, to the appointment. The meal was eaten in the hospital canteen, with access to plates, cutlery and a microwave for heating food.
   3. A researcher guides participant in downloading a free smartphone app (Mandolean.BLE for Android or Mandolean® on iOS). Participant logs into the application using their unique login details.
   4. The app connects to the pre-assigned plate scales via Bluetooth. Once connection is established, the user can open the meal function on the app.
   5. The user chooses the appropriate meal – lunch or dinner – and follows meal instructions displayed on the smartphone screen. A plate is placed on the scale and the user is then prompted to put food on the plate. The Mandolean® records and stores the weight of both the empty and full plate. The user begins the meal, and as they eat and the food is removed from the plate during the meal, its weight decreases, allowing the smartphone app to calculate the rate of food consumption. In addition, at the start of the meal and at regular intervals, a satiety rating scale appears on the smartphone screen and the patient rates her/his level of fullness. The scale has numerical values from 0 (no satiety / very hungry) to 10 (maximum satiety / overly full).
   6. For intervention training: participants randomised to training receive additional training on how to complete an intervention meal. Ahead of this appointment, the researcher assigns a pre-defined meal portion size (grams) and planned meal time (13-15 minutes). The user is instructed that the plate scales will only function when the pre-defined food portion size (total meal) is exact. The user follows the same process as above, but during intervention meals, the user can see their rate of eating in real time compared to an ‘ideal’ eating rate curve. The app also provides real time feedback messages such as ‘Eat a little bit slower’ to remind the user to slow down during the meal. The satiety rating scale also appears during intervention meals. The process for completing the meal is the same.
   7. When the user is finished eating, they press the ‘finish’ button on screen. The data is immediately uploaded to the Mandobase clinical portal, accessible by the research team.
   8. The researcher checks understanding with the user and their parent/carer. Written information is provided to all users (see Supplementary Material Section 2).
