# Supplementary material for: Mobile Health Apps in Pediatric Obesity Treatment: Process Outcomes From a Feasibility Study of a Multicomponent Intervention
Source: JMIR Mhealth Uhealth. 2020 Jul 8;8(7):e16925. doi: 10.2196/16925 (PMC7381070; doi:10.2196/16925)
Supplement: Multimedia Appendix 2 [file mhealth_v8i7e16925_app2.docx]

1. **
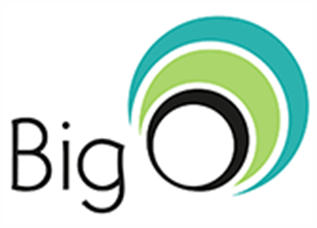
Written standard instructions provided to participants at baseline (all participants) and intervention training (intervention arm only)**

**WHAT I AM DOING WITH THE BIGO STUDY:**

**1. Wear watch every day for 2 weeks**

**2. Wear the watch for 2 nights sleeping each week**

**3. Eat at least 1 meal from the Mandolean every day (lunch or dinner or both)**

**HOW DO I KNOW IF THE WATCH IS WORKING:**

**Check connection with phone before taking off the watch each evening (charge up for few mins if needed):**

**• Ensure Bluetooth is enabled and in wifi / 3G / 4G zone**

**• Check phone and watch are ‘connected’ by opening Wear OS app and in top left hand corner should say ‘connected’ & try changing watch face by choosing different image on Wear OS app – new image should appear instantly on the watch.**

**• Check that BigO app is still installed on the phone & the watch**

Your login details:

Username:

Password:

**What to do:**

- Eat **at least 1 meal per day** using the Mandolean (lunch or dinner or both).
- Choose ‘training’ setting for the meal.

**Using the Mandolean so your meals are saved:**

Your meal may not be saved if you don’t use the scales and app correctly.

Here are instructions for best results:

| **Do** | **Do Not** |
| --- | --- |
| - Keep your plate on the scales for the **whole meal.** - Keep the app open until you are finished eating. - Finish the session completely on the app when you’re finished eating. - Go through the questions at the end of the meal. - Make sure the Mandolean is charged – if the light is red/orange it needs to be charged. | - Take your plate off the scales in the middle of the meal. - Close down the app during your meal. - Play with your phone or open another app during your Mando scales meal. - Close down the app before answering the questions at the end of the meal. |

**How to connect the app to the scales**

1. Open the Mandolean app on your phone
2. Login using details provided
3. Tap menu in the far right corner
4. Choose settings (see picture 1)
5. Turn on the scales & bluetooth
6. Tap connect scales (see picture 2)
7. Scales should connect (see picture 3)


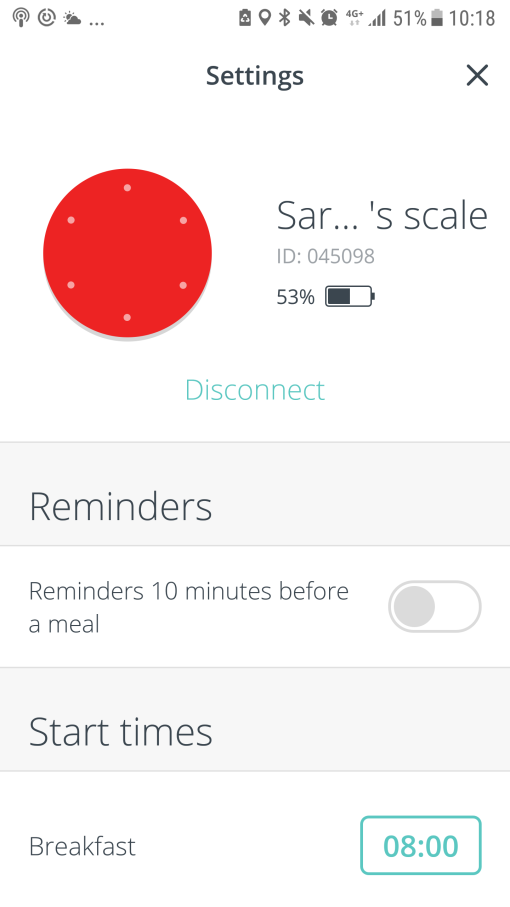

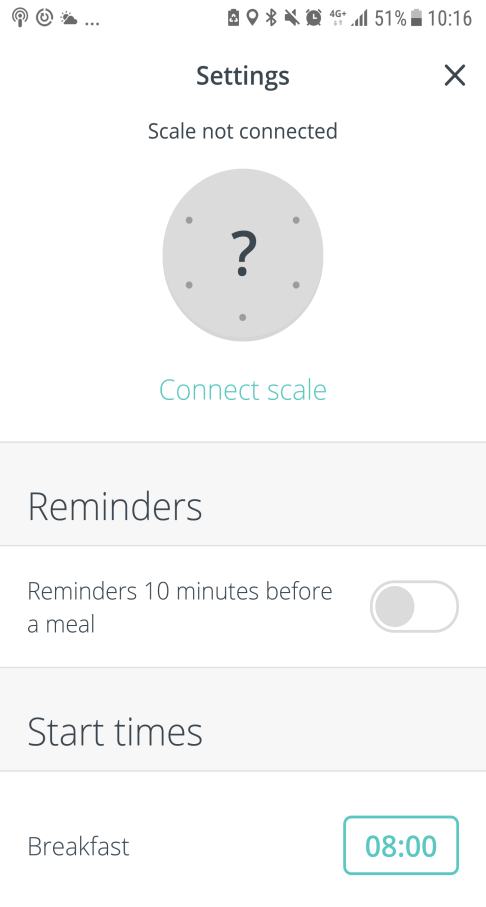

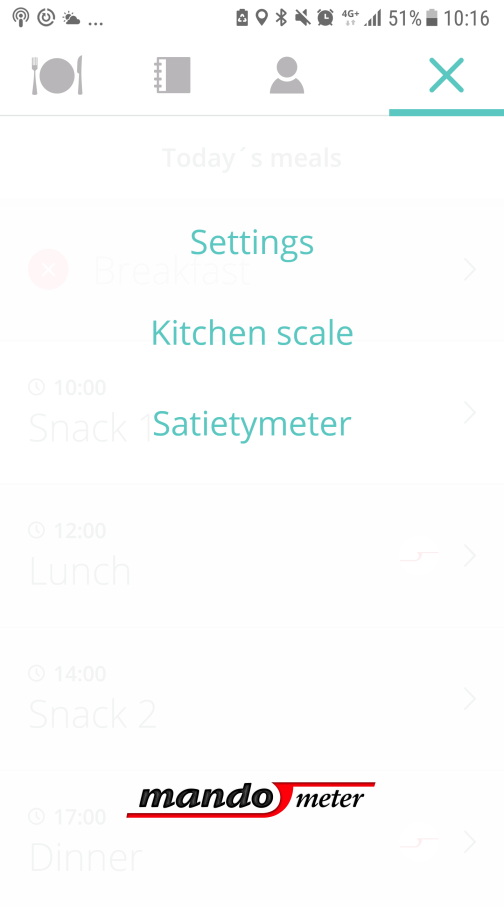


Picture 3: Connected scales

Picture 2: Settings

Picture 1: Main Menu

**How to set up control meal:**

1. **Choose ’today’s meals’ from the top bar icons (see picture 4)**
2. **Choose the meal you are having (lunch or dinner)**
3. **Choose ’control’ (see picture 5)**


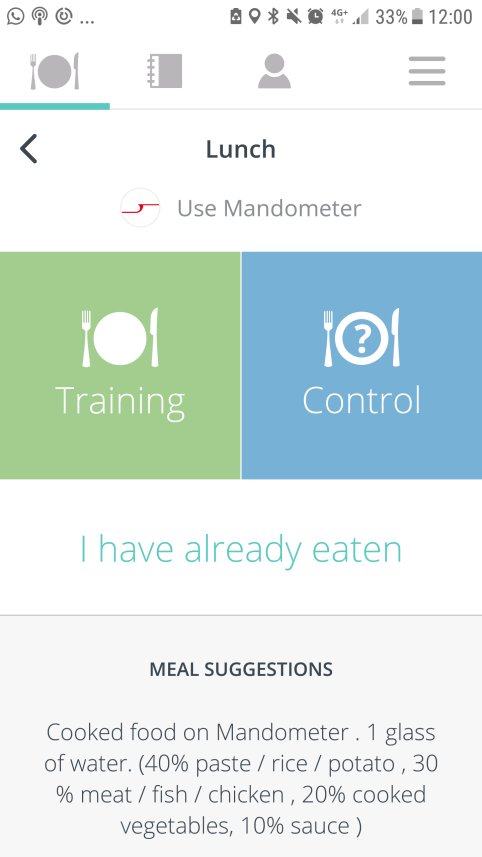

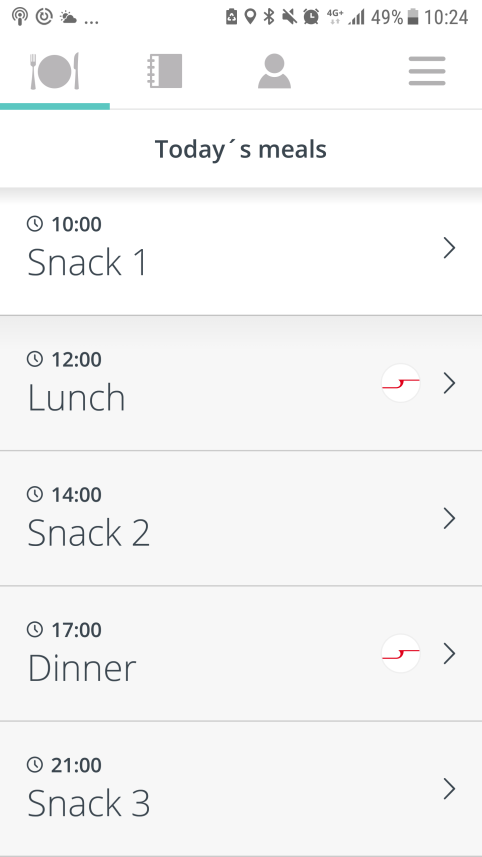


Picture 5: Meal choices

Picture 4: Today’s meals

**How to set up a training meal**

1. **Choose ’today’s meals’ from the top bar icons (see picture 4)**
2. **Choose the meal you are having (lunch or dinner)**
3. **Choose ’training’ (see picture 5)**
4. **Follow instructions on the screen to complete meal.**


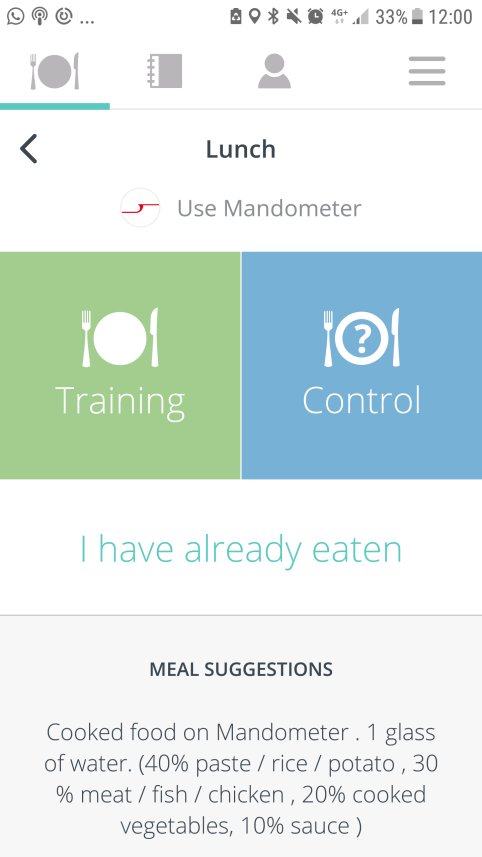

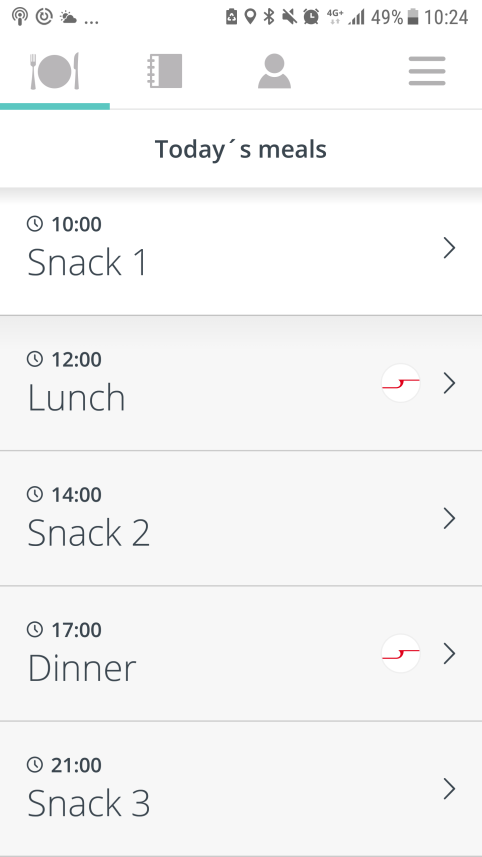


Picture 5: Meal choices

Picture 4: Today’s meals

1. **Follow the instructions on the app for setting up a training meal**
   1. **Place plate on scales, hit continue (see picture 6)**
   2. **Place food on plate – add food until the weight reaches 100% (no more, no less or app won’t proceed with the meal), hit continue/start meal (see pictures 7 and 8)**
   3. **The app will ask you to rate your hunger/fullness on the satiety metre. Do this & hit save (see picture 9)**


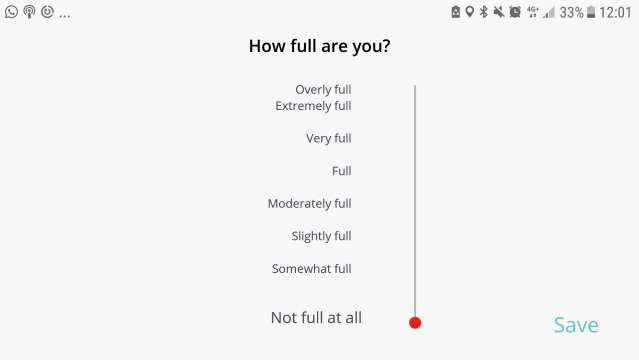

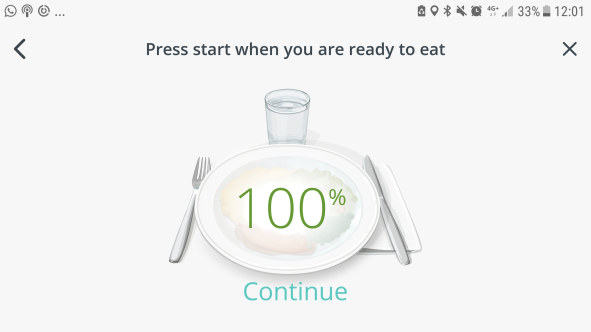

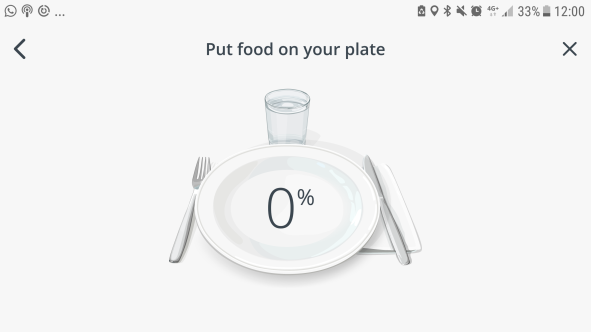

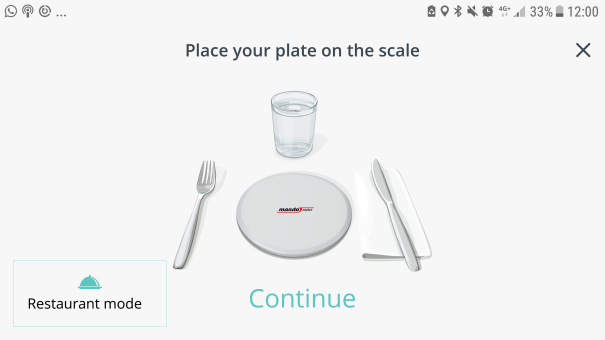


Picture 6: Put plate on scales

Picture 7: Put food on plate

Picture 8: When food at 100% press continue to begin meal

Picture 9: Satiety metre – rate your hunger/fullness

1. Eating the training meal
   1. Eat your meal – follow the instructions on screen during the meal e.g. ’eat a bit slower’ (see picture 10)
   2. When you are finished tap ’x’ in the top right hand of screen. (see picture 11)
   3. Is there any food left on your plate? (see picture 12)
      1. Yes if you didn’t finish the meal completely
      2. No if you’ve cleared your plate
   4. Picture 13 indicates that your training meal has been saved. Well done!

Picture 11: When finished eating ‘x’ out of the graph

Picture 10: Start of training meal and example of instruction from app


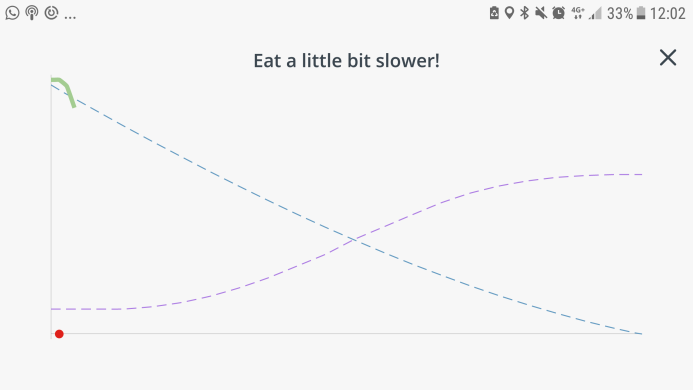

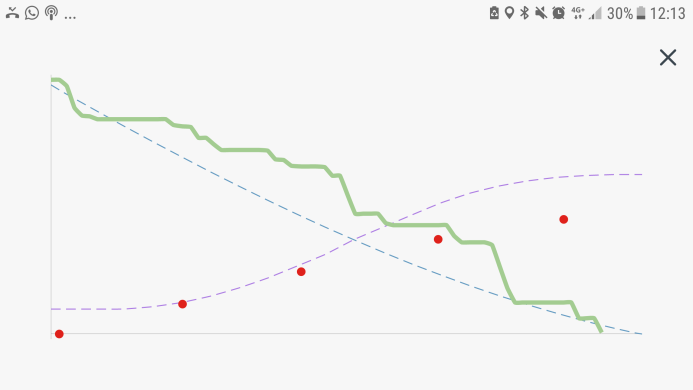


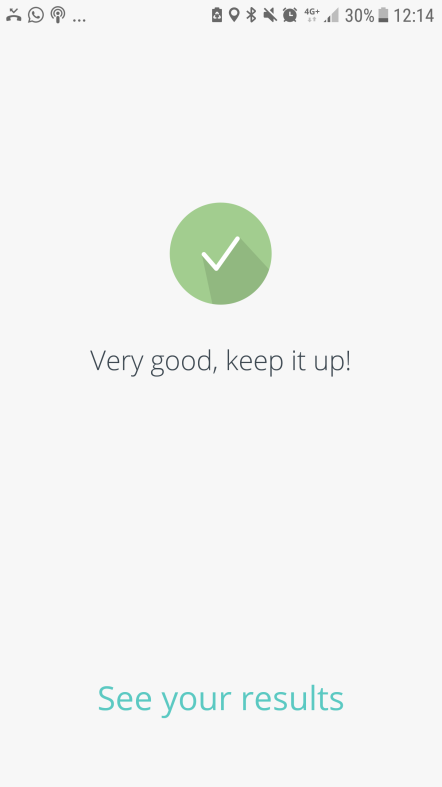

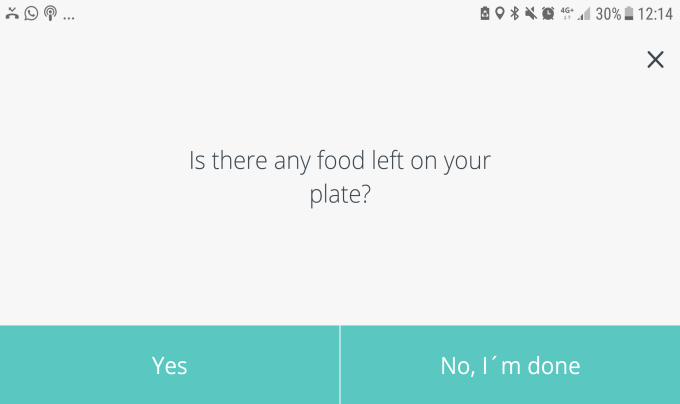


Picture 13: The meal is complete & saved

Picture 12: Answer all questions relating to the meal
