# Supplementary material for: Mobile Health Apps in Pediatric Obesity Treatment: Process Outcomes From a Feasibility Study of a Multicomponent Intervention
Source: JMIR Mhealth Uhealth. 2020 Jul 8;8(7):e16925. doi: 10.2196/16925 (PMC7381070; doi:10.2196/16925)
Supplement: Multimedia Appendix 3 [file mhealth_v8i7e16925_app3.docx]

1. **Description of baseline measurements**

**Patient-level baseline measures pre-intervention included:**

1. Rate of eating measured by Mandolean®; physical activity level measured by smartwatch (Ticwatch E Versions 7.0 and 8.0, Mobvoi) and myBigO app.
2. Anthropometry: Body weight was measured to the nearest 0.1 kg using an electronic scale (SECA, Vogel & Halke, Hamburg, Germany) and height to the nearest 0.1 cm with a stadiometer (SECA, Vogel & Halke) in light clothing and without shoes. Measures were taken in triplicate and mean values calculated. BMI was calculated as weight/height squared (kg/m^2^) and BMI SDS calculated using UK reference data (Cole LMS method).
3. Social, behavioural and emotional functioning was assessed using the Child behaviour Checklist/Youth Self Report (Achenbach & Rescorla, 2001) and self concept by The Piers Harris Questionnaire (Piers & Herzberg, 2002).
4. Quality of life was measured using the PedsQL. (Varni et al., 2001)
5. Self-report eating behaviour was assessed using the Dutch eating behaviour questionnaire (Van Strien et al., 1986).
