# Supplementary material for: Mobile Health Apps in Pediatric Obesity Treatment: Process Outcomes From a Feasibility Study of a Multicomponent Intervention
Source: JMIR Mhealth Uhealth. 2020 Jul 8;8(7):e16925. doi: 10.2196/16925 (PMC7381070; doi:10.2196/16925)
Supplement: Multimedia Appendix 4 [file mhealth_v8i7e16925_app4.docx]

1. **Behaviour Change Techniques incorporated into the intervention**

**Table S1 Behaviour change techniques and sub-techniques linked to implementation actions of the intervention categorised with numbering system according to Behaviour Change Taxonomy (Michie et al., 2013).**

| **Behaviour change Technique** | **Sub-technique** | **Implementation** |
| --- | --- | --- |
| 1 Goals and Planning | 1.1 Goal setting  1.6 Discrepancy between current behaviour and goal  1.9 Commitment | Reduce rate of eating behaviour  Baseline rate of eating compared to target rate of eating  Parents and patients signed informed consent committing to participate in the intervention |
| 2 Feedback and monitoring - | 2.2. Feedback on behaviour  2.6. Biofeedback | In person feedback provided by the research clinician at review appointments  Real-time text on-screen ‘eat a little slower’ provided on smartphone app when exceeding recommended rate of eating during meal times |
| 3 Social support | 3.1 Practical | Parents learned how to set up the Mandolean® with practical meal time demonstration |
| 4 Shaping knowledge | 4.1 Instructions on how to perform the behaviour | Demonstration, verbal and written instructions on using the Mandolean® to reduce rate of eating provided by a trained research dietitian |
| 6 Comparison of behaviour | 6.1 Demonstration of the behaviour | Experience of matching current eating rate to ‘ideal’ rate |
| 8 Repetition and substitution | 8.1. Behavioural practice and rehearsal  8.3 Habit formation | Participant practiced using the technology in demonstration sessions at baseline and randomisation to intervention  Repeated daily use at home over 4 weeks |
| 12 Antecedents | 12.1 Restructuring the physical environment | Incorporating a tool at usual meal time home to slow rate of eating |
