# Supplementary material for: Mobile Health Apps in Pediatric Obesity Treatment: Process Outcomes From a Feasibility Study of a Multicomponent Intervention
Source: JMIR Mhealth Uhealth. 2020 Jul 8;8(7):e16925. doi: 10.2196/16925 (PMC7381070; doi:10.2196/16925)
Supplement: Multimedia Appendix 6 [file mhealth_v8i7e16925_app6.docx]

**Figure: Bar chart showing participants’ socio-economic position for the total group (n=20), intervention (n=8) and control (n=12) groups. Socio-economic position is based on address using a combination of socio-economic predictors in small geographical areas and available from national census data (HP Pobal Deprivation Index for small geographical areas, Central Statistics Office, Dublin, 2016).**
