# Supplementary material for: Mobile Health Apps in Pediatric Obesity Treatment: Process Outcomes From a Feasibility Study of a Multicomponent Intervention
Source: JMIR Mhealth Uhealth. 2020 Jul 8;8(7):e16925. doi: 10.2196/16925 (PMC7381070; doi:10.2196/16925)
Supplement: Multimedia Appendix 7 [file mhealth_v8i7e16925_app7.docx]

1. **Characteristics and participation levels and feedback from children and teenagers who dropped out of the feasibility study before completion.**

**Table S2 Characteristics of non-completers, their treatment group, withdrawal time point, tasks completed and reasons given for withdrawing.**

| **Participant Age and Sex** | **Group** | **Weeks in study** | **Appointments and study tasks complete** | **Reasons given** |
| --- | --- | --- | --- | --- |
| **15.4 yr old**  **girl** | Intervention | 3 | T1, T1a, T2.  Baseline measures: Two intervention meals. | Child reported lack of interest in Mandolean®. |
| **13.1 yr old boy** | Control | 8 | T1, T1a, T2, T3.  Baseline measures, control period. | School and behavioural issues identified by parent as barriers to completing the study. |
| **14.8 yr old**  **girl** | Intervention | 3 | T1, T1a, T2.  Baseline measures. | Participant reported that Mandolean® was too complicated at meal times and didn't want to use it. |
| **13.6 yr old**  **girl** | Control | 2 | T1, T1a.  Baseline measures. | Participant reported lack of interest in the study.  Parent reported child was self-conscious about wearing smartwatch at school. |
| **11.1 yr old boy** | Intervention | 2 | T1, T1a.  Baseline measures. | Parent reported a family illness and could not commit to extra appointments for the study. |
| **15.3 yr old boy** | Intervention | 6 | T1, T1a, T2, T3.  Baseline measures. Did not engage with intervention. | Participant reported a lack of interest in Mandolean® and that using it at meals “…felt like a hassle.” |
| **12.3 yr old boy** | Intervention | 10 | T1, T1a, T2.  Baseline measures, intervention training. Did not engage with intervention. | Parent reported that when they worked away from home, they could not commit to supporting the use of Mandolean® ® regularly. |
| **12.6 yr old boy** | Control | 2 | T1, T1a.  Baseline measures. | Participation delayed owing to illness and then decided to withdraw as child found it was "too much on me". |
