# Supplementary material for: Mobile Health Apps in Pediatric Obesity Treatment: Process Outcomes From a Feasibility Study of a Multicomponent Intervention
Source: JMIR Mhealth Uhealth. 2020 Jul 8;8(7):e16925. doi: 10.2196/16925 (PMC7381070; doi:10.2196/16925)
Supplement: Multimedia Appendix 8 [file mhealth_v8i7e16925_app8.docx]

1. **System Usability Scores for Mandolean and Smartwatch, including qualitative comments recorded by participants and their parents.**

**Table S3 System Usability Scales (SUS) Scores presented for total group, intervention and usual care arms, completers and non-completers and categorised qualitative feedback from participants and their parents.**

| Group | Mandolean®  SUS Score | | | | Smartwatch  SUS Score | | | |
| --- | --- | --- | --- | --- | --- | --- | --- | --- |
|  | n | Mean ±SD | Median | 25^th^, 75^th^ | n | Mean ±SD | Median | 25^th^, 75^th^ |
| Total Group | 14 | 54.1±16.7 | 52.5 | 43.8, 63.1 | 11 | 75.9±18.6 | 77.5 | 57.5, 90 |
| Intervention Arm | 5 | 43.0±18.0 ^a^ | 45.0^c^ | 27.5, 57.5 | 2 | 95.0±7.1^e^ | 95.0 | 90.0, x |
| Usual Care Arm | 9 | 60.3±13.0 ^b^ | 57.5^d^ | 50, 71.3 | 9 | 71.7±17.8^f^ | 75.0 | 53.8, 85 |
| Completers | 9 | 58.1±19.5 | 67.5 | 45.0, 71.3 | 7 | 74.6±17.6 | 77.5 | 57.5, 90 |
| Non-completers | 5 | 47.0±6.8 | 47.5 | 41.3, 52.5 | 4 | 78.1±22.9 | 82.5 | 54.4, 97.5 |
| ^ab^ t-test for equality of means, equal variances assumed p=.059  ^cd^ Independent samples Mann-Whitney U-test, p=.042  ^ef^ t-test for equality of means, equal variances assumed p=.033 | | | | | | | | |
| **Categories of qualitative feedback with representative quotes for the smartwatch and Mandolean®** | | | | | | | | |
|  | **Mandolean®** | | | | **Smartwatch** | | | |
|  | **Connectivity issues**  “It didn’t always connect to the phone easily.” (Participant, 15 year old girl)  “The Mandolean® ® didn't work very well, sometimes halfway through a meal it would stop. And when we go back in to finish meal we couldn't because there was an 'X'. It made a mess of dinner when we had to restart or reload the plate” (Mother, 10 year old girl)  “The Mandolean® ® was very temperamental - the meal timings, disconnecting during a meal, the option to 'continue' not presenting after plate on scales” (Father, 13 year old girl).  **Difficult, awkward or time-consuming**  “Found it awkward transferring food onto plate after Mandolean® ® weighs the plate” (Father of 13 year old boy)  Would change the Mandolean® ® to "set it itself up as it felt like an extra hassle before dinner.” (Participant, 10 year old girl)  “Getting the plate set-up and loading of the food took time and she doesn’t want to wait for it to be ready.” (Mother of 9 year old girl)  **Different family routines a barrier to recommended use.**  School holidays and parents’ shift work: “We were out of routine so fixed meal times didn't work and it was a big factor in not using it” (Father of 13 year old boy)  “You need to have someone home at mealtimes.” (Mother of 9 year old girl)  **General dissatisfaction**  “It was really annoying” (Participant, 10 year old girl)  “I didn’t really like it, it was annoying.” (Participant, 11 year old girl)  “It didn't give any indication of what was wrong, and charging was complicated, and easy to forget. It felt like a hassle.” (Participant, 15 year old boy)  **More aware of eating behaviour**  A number of children and parents reported becoming more aware of their speed of eating as a result of using the Mandolean® ®.  “I would recommend to a friend who eats too large portions too fast” (Patient, 11 year old girl)  **Practical issues**  Dinner plates in one household too heavy for the Mandolean® ® scales so they bought some plastic plates to use with it.  **Forgot**  Some reported regularly forgetting to use at meal times and remembering during the meal, when it was too late to set up. | | | | **Liked it:**  “Normally I bring my phone but I didn't have to because it has the time on it and I like looking at the steps on the watch” (Participant, 11 year old girl)  **Easy to use:**  “The watch was very easy to use and to understand” (Participant, 15 year old girl).  **Battery usage**  “It (watch connected to myBigO app) ran down battery on my phone EXTREMELY fast” (Participant, 15 year old boy).  **Sensory issues:**  Two children found the watch uncomfortable: “It got uncomfortable sometimes” (Participant, 13 year old boy)  **Self-conscious**  One parent of a 15 year old girl contacted the researcher to say that her daughter was self-conscious about wearing the watch at school in case peers asked what it was for. | | | |
